# Supplementary material for: Co-Electrospun Poly(ε-Caprolactone)/Zein Articular Cartilage Scaffolds
Source: Bioengineering (Basel). 2023 Jun 27;10(7):771. doi: 10.3390/bioengineering10070771 (PMC10376865; doi:10.3390/bioengineering10070771)
Supplement: Supplementary file 1 [file bioengineering-10-00771-s001.zip › bioengineering-2459878-supplementary.pdf]

SUPPLEMENTARY MATERIALS

Nanofiber surface of poly(ε-caprolactone)-zein-based one-nozzle electrospun articular cartilage scaffolds

Andre M. Souza Plath [1,*], Stephanie Huber [1], Serena R. Alfarano [2], Daniel F. Abbott [3], Minghan Hu [4], Victor Mougel [3], Lucio Isa [4] and Stephen J. Ferguson [1]

* Correspondence: andre.souzaplath@hest.ethz.ch

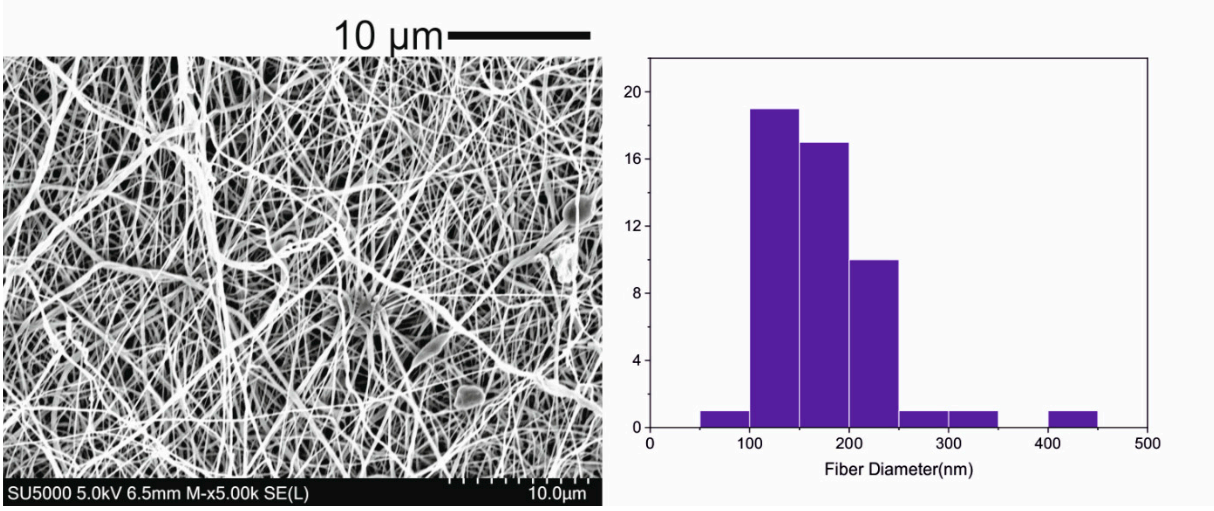

Figure S 1: SEM Images (5000× Magnification) of the electrospun PCL samples in a FA/AA (70/30 vol./vol binary system)

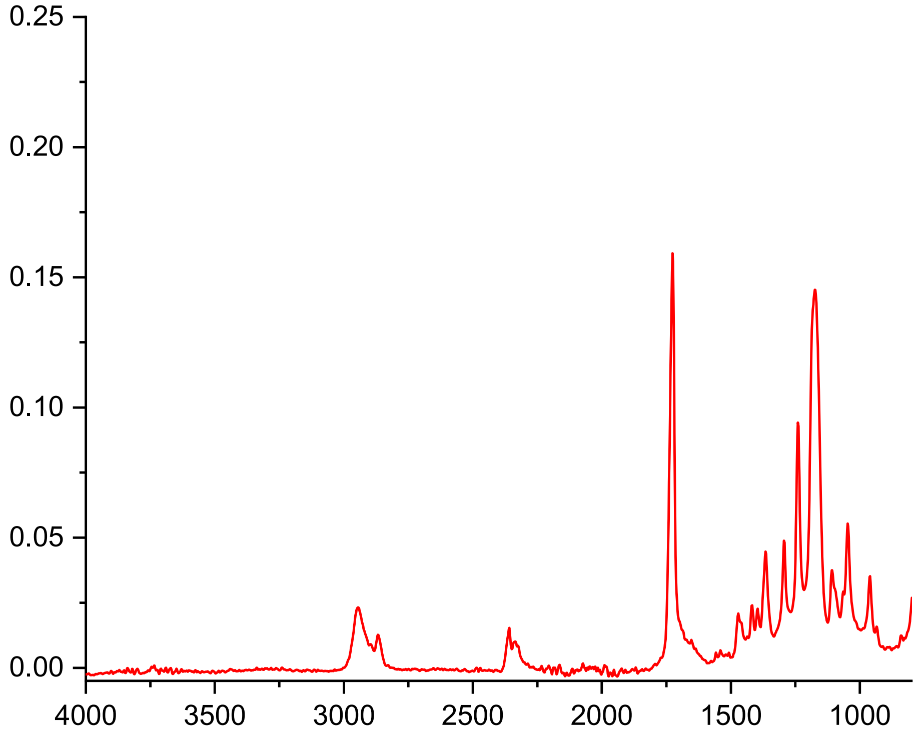

Figure S2: FTIR-ATR of sample 6P50Z incubated overnight in 70% Ethanol at room temperature (32 scans). -COOH and -NH2 stretch, Amide II bands at 3500 cm^{-1} and 1655 cm^{-1} are absent.

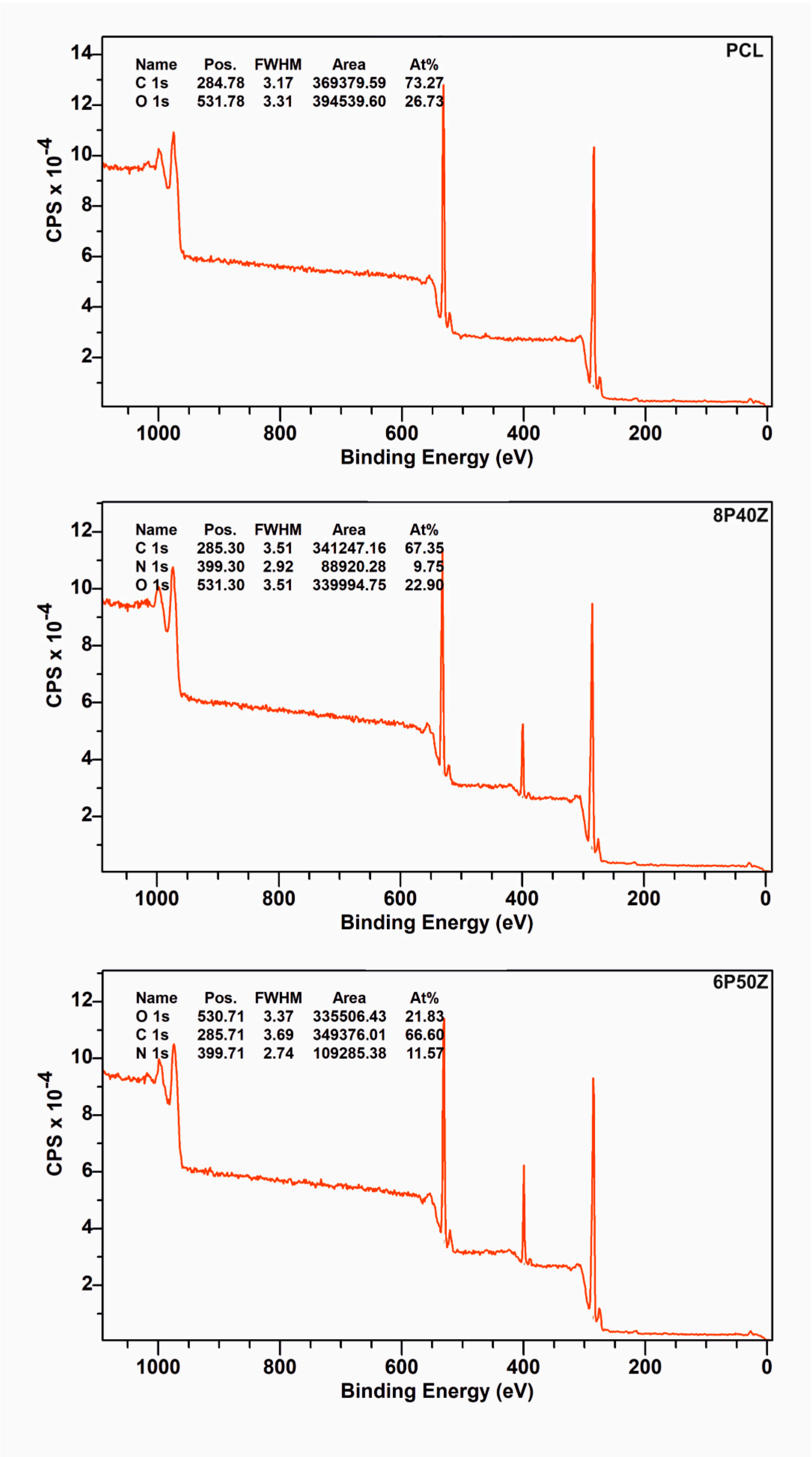

Figure S 3: XPS Survey of electrospun PCL, 8P40Z, and 6P50Z samples. Peak information in Table 3.

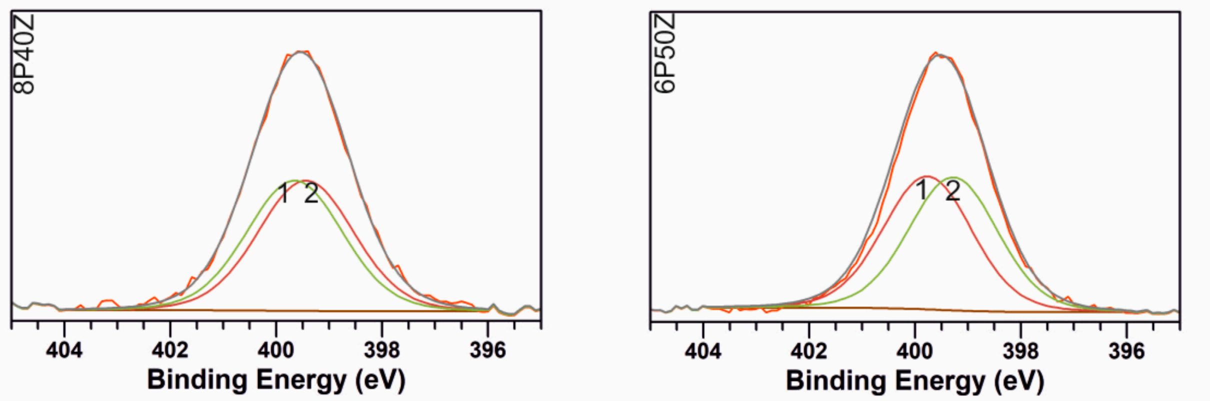

Figure S 4: N 1s high-resolution XPS spectra of 8P40Z and 6P50Z electrospun samples.

	8P40Z BE	8P40Z Peak Area	6P50Z BE	6P50Z Peak Area
1 ($C-NH_3^+$)	399.7	50.0	399.7	50.0
2 (N-C=O)	399.4	50.0	399.3	50.0

Table S 1: N 1s high-resolution spectra of electrospun samples 8P40Z and 6P50Z. BE: Binding Energy.

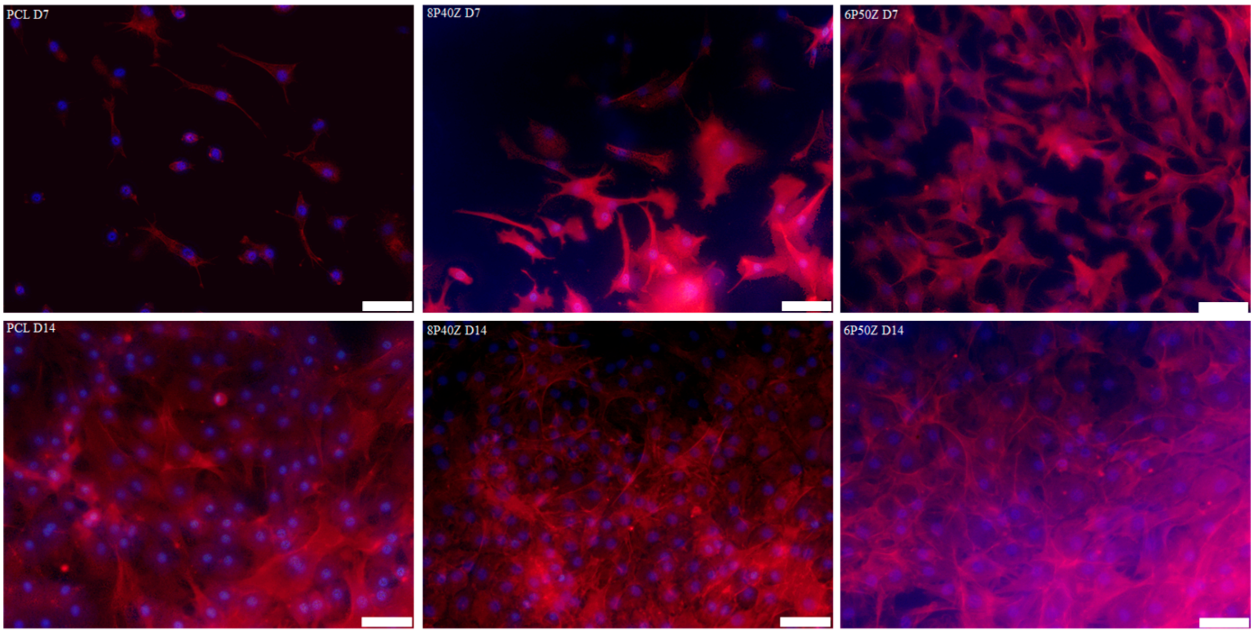

Figure S 5: Widefield Fluorescence Microscopy images obtained with the microscope Olympus IX51 at 20× magnification (scale bars 50 µm)

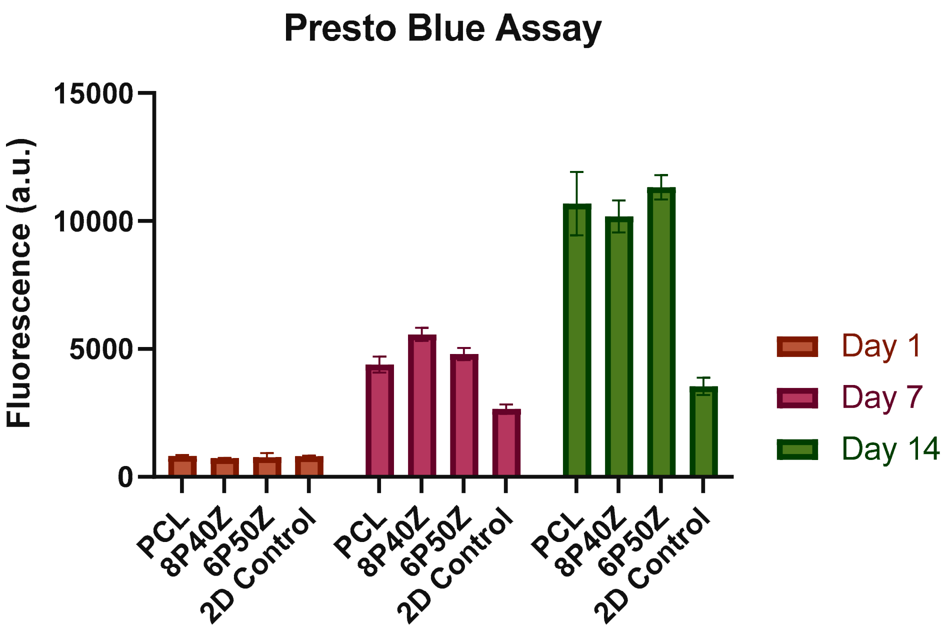

Figure S6: Presto Blue Assay, readings performed at 584nm (n = 12) using a M200 PRO Plate Reader (Tecan Switzerland).
